# Mesenchymal Stromal/Stem Cells and Their Products as a Therapeutic Tool to Advance Lung Transplantation

**DOI:** 10.3390/cells11050826

**Published:** 2022-02-27

**Authors:** Vitale Miceli, Alessandro Bertani

**Affiliations:** 1 Research Department, IRCCS ISMETT (Istituto Mediterraneo per i Trapianti e Terapie ad alta specializzazione), 90127 Palermo, Italy; 2 Thoracic Surgery and Lung Transplantation Unit, IRCCS ISMETT (Istituto Mediterraneo per i Trapianti e Terapie ad Alta Specializzazione), 90127 Palermo, Italy

**Keywords:** lung transplantation, ex vivo lung perfusion, mesenchymal stromal/stem cells, cell therapy, lung preservation, lung reconditioning

## Abstract

Lung transplantation (LTx) has become the gold standard treatment for end-stage respiratory failure. Recently, extended lung donor criteria have been applied to decrease the mortality rate of patients on the waiting list. Moreover, ex vivo lung perfusion (EVLP) has been used to improve the number/quality of previously unacceptable lungs. Despite the above-mentioned progress, the morbidity/mortality of LTx remains high compared to other solid organ transplants. Lungs are particularly susceptible to ischemia-reperfusion injury, which can lead to graft dysfunction. Therefore, the success of LTx is related to the quality/function of the graft, and EVLP represents an opportunity to protect/regenerate the lungs before transplantation. Increasing evidence supports the use of mesenchymal stromal/stem cells (MSCs) as a therapeutic strategy to improve EVLP. The therapeutic properties of MSC are partially mediated by secreted factors. Hence, the strategy of lung perfusion with MSCs and/or their products pave the way for a new innovative approach that further increases the potential for the use of EVLP. This article provides an overview of experimental, preclinical and clinical studies supporting the application of MSCs to improve EVLP, the ultimate goal being efficient organ reconditioning in order to expand the donor lung pool and to improve transplant outcomes.

## 1. Introduction

Lung transplantation (LTx) has become the treatment of choice for patients with end-stage respiratory failure and, over the past decades, the worldwide survival of lung transplant patients has increased significantly [1]. Unfortunately, the short- and long-term outcomes of LTxs are still less favorable than other solid organ transplants, the main faults being organ shortage and the fact that more than 80% of potential organ donors are not suitable or used for transplantation [2,3,4,5,6]. Moreover, other factors, such as postoperative graft dysfunction (PGD), infections, and rejection may also contribute to post-transplant mortality [7,8].

Although the use of extended-criteria lung donors and donors after cardio-circulatory death is increasing, there is still wide consensus in the transplant community that a large proportion of potentially viable grafts is discarded due to fear of early graft dysfunction and the increased postoperative morbidity and mortality of the recipient [1,3,9,10]. Ischemia-reperfusion injury (IRI) is the leading cause of early postoperative lung dysfunction, and is characterized by alveolar damage and lung edema usually occurring within 72 hours after LTx [11]. These pathological processes are driven by both oxidative stress and inflammatory pathways, which cause significant damage to the lung parenchyma, leading to the development of early PGD and consequent chronic lung allograft dysfunction [1,10,11,12]. Therefore, a reduction in these adverse effects could significantly improve the success of LTx.

Recently, it has been shown that the use of normothermic ex vivo lung perfusion (EVLP) may be helpful in increasing the number and improving the results of lung transplantation [13,14]. This procedure provides the opportunity to evaluate donor lung function, and different treatments can also be used to further reduce IRI [15,16,17]. Therefore, the improvement of this technique represents a promising strategy to increase the number of suitable organs and to advance LTx.

A suitable approach to improving EVLP technique consists of using mesenchymal stromal/stem cells (MSCs) and/or their secretome, which contains extracellular vesicles (EVs, such as exosomes and EXOs) and other bioactive molecules, including cytokines, chemokines, growth factors, angiogenic and immunomodulatory factors [18,19,20,21]. Although the mechanisms are not fully defined yet, it has been widely demonstrated in both preclinical and clinical studies that therapeutic effects of MSCs are mediated, at least in part, by paracrine factors [21,22,23,24]. MSCs’ therapeutic properties have been also evaluated in a phase II clinical trial to treat acute respiratory distress syndrome (ARDS) [25]. MSCs and/or their products are able to mitigate both lung injury and inflammation in different experimental models, and the infusion of MSCs protects transplanted lungs from IRI [26,27,28,29]. Moreover, it has been found that MSC-based treatment during EVLP is associated with a decrease in ischemic injury of human donor lungs [30,31,32]. In this case, to avoid the founding of infused MSCs in the lung parenchyma, cell-free therapies, including the use of MSC-derived EXOs and MSC-derived conditioned medium (CM), could be considered new approaches to obtaining MSCs’ beneficial effects. EXOs are nanosized structures carrying functional molecules [33], and can be considered a promising therapeutic tool for acute lung injury because they reduce inflammation and enhance tissue regeneration [34,35,36]. Similarly, MSC-derived CM has demonstrated beneficial effects on lung diseases [37,38,39,40,41,42,43]. The use of MSC-derived products rather than direct use of MSCs can prevent all the risks associated with live-cell transplants. Therefore, the use of these products is emerging as a promising approach in the field of LTx. In this review, we describe the therapeutic properties of MSCs and their products as promising tools to improve EVLP techniques, with the ultimate goal of advancing LTx outcomes.

## 2. Current Challenges in Lung Transplantation

Lung transplantation has become the gold standard treatment for patients with end-stage lung disease. According to recent data, approximately 4,600 transplants are performed every year worldwide, and 80% are bilateral [44]. Although the results of LTx have significantly improved during the past twenty years, morbidity and mortality remain high when compared with other solid organ transplants [6,44]. One central issue in lung transplantation is the chronic shortage of donors. This is related to both the donor pool (which should be expanded) and the need for better lung preservation techniques to convert a usually unsuitable organ into an organ exploitable for transplantation. Therefore, our understanding of lung biopathology and the implementation of new techniques/treatments capable of expanding the lung donor pool will have a significant impact in the field.

### 2.1. Donor Shortage and Expansion of Donor Pool

Although the number of LTxs worldwide has been steadily increasing over the past decade, there is still a significant imbalance between the number of available donors and patients’ needs, and mortality on the waiting list is still high. An improvement was achieved thanks to the introduction of the lung allocation score (LAS), which was implemented in the United States in 2005 and is still under refinement [45]. The LAS significantly decreased deaths on the waiting list without affecting overall post-transplant outcomes [46]. However, the number of patients dying on the waiting list is still high, and the shortage of grafts persists [47]. This scenario is not only due to the shortage of available donors, but also to the low utilization rate of donor lungs. Indeed, it has been evaluated that a large number of multiorgan donors are not suitable for LTx [3,13] and, in particular for donation after circulatory death (DCD), lungs are considered eligible for transplantation in only 2% of cases [13]. Potentially suitable lungs are deemed ineligible for transplant due to various criteria, including the donor history, pneumonia, chest trauma, aspiration, purulent secretions, positive sputum gram stain, or inadequate gas exchanges [1,48,49]. This long list makes it clear why only 15–20% of donated lungs are allocated. During the last decade, the use of extended donor criteria, including DCD donor-derived lungs, has emerged as a promising approach for increasing the donor pool. Controlled and uncontrolled DCD lung donation has emerged as a promising strategy to retrieve viable lungs for transplantation, although the legislation on declaration of death can vary in different countries and can pose a significant limitation to this process [48]. The use of DCD in LTx has been met with growing interest following the suggestion in different reports that up to 30% more donors could be recruited by this approach [50,51,52]. Indeed, although there is no general consensus on the use of DCD lungs (because of the potential for lung damage resulting from this strategy), the growing scientific evidence has demonstrated similar or superior outcomes with DCD donor lungs compared to lungs derived from brain dead patients [48,53]. Interestingly, in the case of uncontrolled DCD (where the donation is made after unexpected out-of-hospital cardiac arrest), due to both the prolonged warm ischemia time post-mortem and the inability for pre-procurement assessment of the lungs, the use of EVLP is strongly recommended before LTx [54]. In this case, EVLP becomes crucial, because it allows the assessment of the lungs and facilitates timing and logistics for LTx management [55]. The combined use of uncontrolled DCD with EVLP in LTx may represent a useful strategy to successfully expand the donor pool.

### 2.2. Lung Preservation by Ex Vivo Lung Perfusion (EVLP)

Ex vivo lung perfusion (EVLP) is a promising technique for the evaluation and recovery of compromised donor lungs. Following preliminary results produced since 1990 by Steen and collaborators [56,57], the Toronto lung transplant group, in 2011, demonstrated that EVLP is feasible in clinical practice [58]. During EVLP, the lungs are placed into a device providing ventilation and perfusion for 4–6 h [58,59,60]. EVLP allows both the evaluation of lungs outside of the donor and of the organ preservation through administration of therapeutic treatments. Recently, Tikkanen et al. demonstrated that EVLP is a safe and effective strategy for assessing high-risk donor lungs before LTx. Furthermore, they showed that with the use of EVLP, long-term survival, graft function, and improvements in quality were comparable to those of conventionally selected donor lungs [61]. Currently, although the EVLP technique has the potential to increase the number of usable lungs up to 30% [58,60], it is not routinely used in clinical practice for lungs accepted for standard donation; rather, it is considered a tool to allow for further evaluation of lungs that are deemed to be of low quality. However, there is growing evidence that EVLP could also serve for normally accepted lungs. EVLP can reduce ischemia-reperfusion injury (IRI), with the potential to improve organ quality and to reduce primary graft dysfunction (PGD) [62]. Interestingly, the idea of EVLP as a therapeutic platform to reduce inflammation, edema, and infection in allografts is very promising. For instance, in a swine model of DCD donor lungs, it has been shown that treatment with adenosine A2A receptor agonist during EVLP allows prolonged cold preservation of lungs [63]. Cypel et al. demonstrated that during EVLP, the delivery of an IL-10 gene with an adenoviral vector was able to improve lung function in injured human donor lungs [64]. Recently, Nakajima et al., in a swine model of IRI and EVLP, revealed that the administration of MSCs in donor lungs during EVLP ameliorated IRI both during perfusion and after transplantation [65]. In the next sections, we propose the therapeutic properties of MSCs as an important tool for the improvement of LTx by enhancing EVLP performance.

## 3. Mesenchymal Stromal/Stem Cells (MSCs) and Their Therapeutic Effects

MSCs exist in adult tissues, such as fat [66], bone marrow (BM) [67], dental pulp (DP) [68], and neonatal tissues, including umbilical cord (UC) [69] and placenta tissue [70], where they participate in the maintenance of stem cell niches and tissue homoeostasis [71]. MSCs are mainly obtained from BM, though other sources such as neonatal tissues are commonly used to obtain MSCs from both umbilical cord and amniotic membrane [72,73,74,75]. In BM, only 0.001 to 0.01% of the cells are MSCs [76]. Although a comparable number of MSCs can be found in neonatal tissue, this cell population shows better expandability in vitro, probably because of their fetal nature, as remarked by the expression of pluripotency markers and by a higher multilineage differentiation capacity [73,75,77,78]. Neonatal MSCs showed comparable regenerative effects to bone-marrow-derived MSCs (BM-MSCs) [73]. In addition, the use of neonatal-tissue-derived MSCs has some advantages such as being obtained easily in higher quantities without invasiveness, and being readily cultured to a sufficient number for their use.

### 3.1. Biological Role of MSCs

From a functional point of view, the scientific community presumes that MSCs belong to subsets of stem/progenitor cells in a specific microenvironment termed the “stem cells niche”. In this case, these cells support tissue regeneration in both physiologic and pathologic conditions, contributing to tissue homeostasis [21,79,80,81]. MSCs are able to produce a plethora of trophic/regulator factors such as proteins and EXOs, and orchestrate individual components of the tissue microenvironment by differentiating or attracting supporting cells to a niche [21,79,80,81,82]. Moreover, numerous in vitro and in vivo studies have shown that MSCs are able to perform homing/migration and immunosuppression during pathophysiological processes [83,84,85,86,87]. For these reasons, MSC therapy has been extensively investigated to evaluate its therapeutic efficacy in degenerative and/or inflammatory diseases [88,89,90,91,92].

### 3.2. Therapeutic Properties of MSCs

The therapeutic properties of MSCs have been examined in both preclinical [88,90] and clinical settings for the treatment of various disorders, including cardiovascular, neurodegenerative, immune, lung, liver, kidney and orthopedics diseases (clinicaltrials.gov). Due to low expression of CD40, CD80, CD86, and major histocompatibility complex I and II (MHC I/II) [93,94,95], MSCs are immuno-privileged; this feature make these cells a useful tool to be applied in the field of cell therapy.

MSCs possess immunomodulatory [84,96], trophic [97], angiogenic [98] and anti-oxidative properties [99], and all of these effects appear to be mediated, at least in part, by the production of a functional secretome. Indeed, in many in vitro and in vivo disease models, MSC-derived products have been identified as responsible for therapeutic effects [100]. These cells can produce many cytokines/chemokines controlling the immune system by interacting with both innate and adaptive responses, resulting in immunosuppression and the induction of tolerance [101]. It has been shown that MSCs secrete crucial anti- and pro-inflammatory factors (PGE2, Indoleamine 2,3-dioxygenase, TGFβ1, TSG-6, HGF, IL-10, IL-6, IFNγ, TNF-α, LIF), which, depending on their ratio, regulate the pro- or anti-inflammatory activity of MSCs. In addition, final immunoregulatory properties may be influenced by cell culture conditions that can prime/enhance MSCs’ properties [21,84,102,103,104,105,106].

MSCs also have the capacity to migrate to injured tissues, contributing to tissue repair/regeneration [107]. In this case, these cells put in place active regulation by producing paracrine components that impact on tissue survival, repair and regeneration, and also by activating tissue resident stem cells [21,108,109]. The secretion of numerous soluble factors has been considered responsible for the pro-angiogenic and anti-apoptotic effects of MSCs [98,105]. Apoptosis is known to occur during IRI, playing a crucial role in the IRI-dependent impairment of organ function [110]. It has been shown that injection of MSCs in a cardiomyopathic animal model decreases the level of apoptosis by increasing the Bcl-2/Bax ratio and inhibiting the level of activated caspase 3 [111]. CM derived from both BM-MSCs and adipose-derived MSCs (AdMSCs) was shown to be able to improve cell viability, to reduce the secretion of pro-inflammatory factors and to enhance IL-10 anti-inflammatory cytokine production in hypoxic injured rat alveolar epithelial cells. These observed effects were due to the inhibition of p38 MAPK phosphorylation and the enhancement of Bcl-2 expression, promoting repair and cellular survival [43]. Moreover, it has been demonstrated that CM derived from human amnion-derived MSCs (AMSCs) was able to preserve the viability and delay apoptosis in A549 human alveolar epithelial cells, throughout the down-regulation of inflammatory factors and the upregulation of anti-apoptotic factors [41]. BM-MSC-derived CM supports lungs to attenuate ischemia by inhibiting pro-inflammatory cytokines [39], and AdMSC-derived CM protects the mouse liver from IRI [112]. An important functional component of MSC-derived CM is represented by EVs, such as EXOs. MSC-released EXOs can be transferred into the cells [113]. In this case, the transfer of functional biomolecules, including mRNAs, miRNAs and protein, can regulate/protect target cells [114,115]. It has been revealed that in an in vitro model, the suppression of T cells by MSCs was partly mediated via EVs [116], and that MSC-derived EXOs can transport miRNAs, showing an immunomodulatory capacity [84]. Also in an in vivo rat model, it has been shown that the injection of MSC-derived EVs protects the kidneys from IRI [117].

Oxidative stress is a pathological process common to cellular/tissue injury and inflammation, and is involved in many pathological processes including IRI [99]. Growing evidence derived from both in vitro and in vivo studies supports that MSCs can exert anti-oxidant properties by scavenging free radicals, promoting endogenous anti-oxidant defenses, altering mitochondrial bioenergetics, and donating functional mitochondria to damaged cells [99].

The above-mentioned properties may explain, at least in part, the cytoprotective and anti-inflammatory potential of MSCs. Overall, the multiple ability of MSCs to regulate many pathological processes offers a rationale for the use of MSCs as a promising therapeutic tool.

## 4. Mesenchymal Stromal/Stem Cell (MSC)-based Therapeutic Approaches to Improving Lung Transplantation

Survival rates after LTx have improved, yet outcomes are still poorer than other solid organ transplants [3,6]. The advances of the lung preservation techniques and the better understanding of the main mechanisms governing IRI processes can lead to a significant reduction in the incidence of lung PGD [118]. This condition is a type of acute lung injury that results from IRI and represents the major cause of early post-transplant morbidity and mortality [119]. In recent years, it has been shown that MSCs are able to reduce PGD. Jarvinen et al. showed that human lung resident MSCs have the potential to modulate immunological responses [120], and McAuley et al. revealed that MSCs can have the ability to restore alveolar fluid clearance in human lungs rejected after transplantation [32]. Moreover, allogenic MSCs were able to reduce both acute lung injury (ALI) in an animal model and CLAD in human lung transplant recipients [121,122]. In fact, much scientific evidence has revealed the therapeutic properties of MSCs in different lung disease models [123,124,125,126,127,128,129,130,131,132,133,134,135,136,137,138], including those induced by IRI-related pathological processes. In Table 1 we summarized both in vitro and in vivo studies reporting the use of MSCs and/or their products in preventing lung injury/dysfunction. For LTx, there is a clinical need to implement new strategies for the prevention and treatment of IRI, and MSCs and/or their products represent a new therapeutic tool to prevent the occurrence of unwanted complications to further improve the success of LTx.

### 4.1. Therapeutic Effects of MSCs on Ischemia-Reperfusion Injury (IRI)

Ischemia-reperfusion injury events can occur in the case of stroke or myocardial infarction, as well as in solid organ transplantation. Prolonged ischemia causes deprivation of both oxygen and nutrients, leading to cellular metabolic and ultrastructural pathological changes [142]. Pathological effects mediated by IRI are often potentiated by the onset of inflammation processes, affecting organ quality and transplant outcomes in solid organ transplantation [143]. In this case, IRI-associated injury can be attenuated by cold ischemia storage, but it cannot be completely prevented [144]. Moreover, oxidative stress is a crucial process of IRI-associated effects, leading to the production of toxic molecules [142]. Much scientific evidence shows that MSCs have been able to decrease inflammation and IRI in both in vitro and in vivo models, and these effects are mediated by different mechanisms, including paracrine activity producing a functional secretome [145]. It has been shown that AdMSCs were able to reduce myocardial IRI and to decrease pro-inflammatory cytokine in an in vivo mouse model [146]. In an IRI rat model, Cui et al. demonstrated that AdMSC-derived EXOs were able to protect the myocardium [147]. Using a rat model of acute renal failure, Mias et al. demonstrated that an injection of BM-MSCs protected the kidneys from IRI, and this effect was enhanced when the cells were primed with melatonin. That result appears to be mediated, at least in part, by the production of soluble factors such as b-FGF and HGF [148]. Additionally, in liver IRI models, some therapeutic effects of MSCs can be attributed to soluble factors. Indeed, in a mouse model of liver IRI, it has been shown that the injection of BM-MSC-derived EVs decreased both inflammation and IRI, also reducing cell apoptotic levels and the production of ROS [149]. In a different in vivo model of liver IRI, an injection of BM-MSCs ameliorated hepatic IRI by reducing inflammation and liver apoptosis, and by increasing antioxidant proteins [150,151]. Beneficial effects of MSC treatments were also showed in different lung IRI models. Sun et al. revealed that the autologous transplantation of AdMSCs reduced ischemia-reperfusion lung injury in a rodent model [139]. Lu et al., in a rat model of lung IRI, showed that an intravenous injection of BM-MSCs reduced both pulmonary edema and pro-inflammatory factors, while increasing anti-inflammatory factors [152]. Moreover, MSC therapy may reduce acute IRI and fibrotic responses in human lungs rejected for transplantation, preventing the severity of PGD [32].

### 4.2. MSCs as Therapeutic Tool to Improve EVLP

Focusing on expanding the donor pool to reduce the mortality of lung transplant in recipient patients, different approaches are currently being explored, including the extended criteria for the selection of donors [153], and lung procurement from donors after cardiac death [3]. These strategies have led to the use of the so-called “marginal” organs that do not fulfill the standard criteria of donor lungs. For this scenario, the ex vivo lung perfusion (EVLP) technique was developed in order to: 1. assess graft function after procurement and before implantation; 2. preserve the graft after harvesting; and 3. repair/regenerate potential grafts previously considered unsuitable for transplantation [17,154]. In a lung-transplanted rat model, EVLP treatment was able to protect lung tissue against IRI side effects [155] and, to further reduce IRI complications, pharmacological treatments are possible either by intravascular or endobronchial administration [15,16]. Moreover, although the molecular mechanisms underpinning lung regeneration during EVLP have not been explored yet, recent studies have analyzed metabolic/proteomic events to reveal potential regenerative effects of EVLP [156,157]. This may offer an additional opportunity to evaluate both questionable donor lungs and the effectiveness of new treatments.

In recent years, promising advances in the field of MSC therapy have been made, and the use of MSCs has also become a potential clinical tool to treat complex lung diseases [158]. MSCs can secrete crucial paracrine factors that regulate endothelial/epithelial permeability, attenuate inflammation and potentiate anti-microbial action in lungs [141,159]. These characteristics, together with the fact that MSCs possess anti-oxidant properties [99], have led researchers to investigate biological treatments based on the use of MSCs and/or their products also in the field of lung transplantation. In particular, MSC-based treatments have been tested to improve EVLP and, while some studies have directly used MSCs, similar results have been achieved using MSC-derived products such as EVs and CM. La Francesca et al. showed that BM-MSCs were able to decrease cold ischemic injury in ex vivo perfused donor lungs. In particular, in this pilot study, two effects were observed: a significant reduction in pro-inflammatory cytokines, and an increase in anti-inflammatory cytokines in non-transplantable human lungs [30]. Mordant et al. showed that, in a swine lung IRI model, UC-MSC treatment administered during EVLP was able to attenuate IRI by improving the efficacy of EVLP. This effect appears to be attributable to increased levels of VEGF and a decreased concentration of circulating IL-8 [140]. In a similar study model, Nakajima et al. showed that MSCs were able to attenuate ischemic injury in donor lungs both during EVLP and after transplantation. In this case, the observed effects appear to be ascribed to both the increase in HGF and IL-4, and the decrease in TNFα and cell-death markers [65]. On the other hand, Lonati at al., in a rat lung IRI model, used MSC-derived EVs administered during EVLP, showing that this product was able to improve lung tissue integrity, decreasing the vascular resistance and up-regulating crucial genes involved in the resolution of both inflammation and oxidative stress [34]. Miceli et al., in an in vitro model of human lung IRI and EVLP, showed that AMSC-derived CM was able to attenuate IRI effects by improving the efficacy of EVLP, increasing anti-inflammatory factors and up-regulating anti-apoptotic factors [41]. Gennai et al. showed that BM-MSC-derived EVs were able to increase the alveolar fluid clearance in human donor lungs rejected for transplantation and perfused with EVLP [141]. Moreover, both BM-MSCs and BM-MSC-derived CM, when administered during EVLP, were able to increase alveolar fluid clearance in human lung injured by *E. coli* endotoxin [31]. UC-MSCs and UC-MSC-derived EVs, in a mouse lung IRI model, were able to attenuate lung dysfunction and injury by improving the efficacy of EVLP, and these effects were attributable to a decrease in edema, neutrophil infiltration, and pro-inflammatory cytokines, and increase in KGF, PGE2 and IL-10 [35].

**Figure 1:**
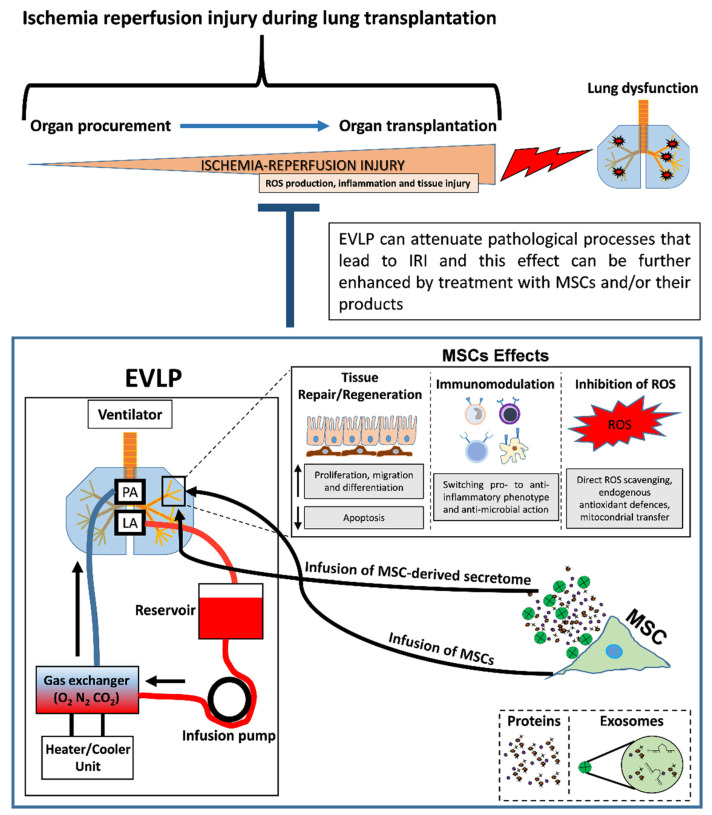
Therapeutic strategies to improve ex vivo lung perfusion (EVLP) during lung transplantation. EVLP treatment may sometimes be not sufficient to mitigate ischemia-reperfusion injury (IRI) effects, and lung graft can reveal the lung to have a poor quality after transplantation. The treatment with mesenchymal stromal/stem cells (MSCs) and/or their products (conditioned medium and/or exosomes containing growth factors, cytokines and chemokines) can effectively potentiate EVLP performance and attenuate IRI adverse events by inducing: 1. tissue repair/regeneration; 2. inhibition of reactive oxygen species (ROS); 3. immunomodulation. These effects can lead to the improvement of clinical outcomes after lung transplantation.

Therefore, the overall data show that the role of MSCs in improving lung recovery appears to be mediated mainly by MSC paracrine signaling; furthermore, the use of MSCs and/or their products, including EVs and CM, represents a promising approach to improving EVLP reconditioning of transplanted lungs. Because of MSCs ability to secrete paracrine factors with anti-inflammatory, anti-oxidant and anti-apoptotic properties, MSCs and/or their products represent a useful tool for integration with EVLP to further preserve/regenerate lung tissue by attenuating IRI during LTx (Figure 1).

## 5. Conclusions and Perspectives

Many preclinical studies in animal models have clearly shown that the therapeutic effects of MSCs on lung injury, and specifically on ischemia-reperfusion injury after LTx, are mediated by the reduction in inflammation [26,27,28,29,34,35,36,37,39,40,65,125,129,137,140]. Moreover, in a preclinical setting, it has also been demonstrated that MSCs have been able to protect human lungs during transplantation [30,31,32,141]. Interestingly, these effects appear to be related, at least in part, to the paracrine secretion of MSC factors, including proteins and EVs (such as EXOs), which offer an opportunity to develop new cell-free treatments for human lung diseases. On the other hand, only two clinical studies (clinicaltrial.gov, NCT number: NCT04714801 and NCT02181712) are currently testing the potential role of MSC therapy in lung transplantation to avoid graft rejection. Therefore, although many works have shown the potential application of MSCs in improving the outcomes of lung transplantation, these effects have not been confirmed in appropriate clinical studies yet. Several issues can explain the limited use of MSCs in this field. In our opinion, there are still relevant limitations to the use of MSC therapy in lung transplant clinical practice. MSCs display mutable properties related to the biological and technical variability of their preparation, and these may impact on the therapeutic potency of MSCs [21,88,95]. In particular, the optimal source, dose, and priming of MSCs is crucial and should be optimized. The best EVLP strategy and protocols to treat lungs before transplantation still need to be elucidated. In addition, another important aspect to be clarified is whether the use of MSC-derived products, such as secretome and/or EXOs, can offer additional benefits compared to standard preparations. The understanding of these critical issues could allow the introduction of MSC treatments in clinical lung transplantation. More efficacy studies—for example, in large animals—are needed to determine the best approach for a human clinical setting. Further investigation is needed to better integrate MSC therapy with EVLP use. This new strategy can lead to enhanced EVLP performance, with the ultimate goal of increasing the number of donor lungs and their quality. Moreover, the routine use of EVLP in clinical practice can favor an improvement in lung transplant management and outcomes.

## Figures and Tables

**Table 1:** Summary of studies reporting the use of MSCs and/or their products in preventing lung injury.

Use of Cells or Their Products	Study Model	Effects Due to MSC Treatment	Mechanisms	References
BM-MSCs	Mouse lung IRI	Protection against cold IRI in lung transplants	Improved arterial blood oxygenation capacity, reduced levels of pro-inflammatory cytokine and cell apoptosis	[29]
MSC-derived EVs	Rat lung IRI and EVLP	Improved tissue integrity and metabolism	Decrease in vascular resistance and rise in perfusate NO metabolites; Up-regulation of genes involved in the resolution of both inflammation and oxidative stress	[34]
UC-MSCs and UC-MSC-derived EVs	Mouse lung IRI	Attenuation of lung dysfunction and injury by improving the efficacy of EVLP	Decreased levels of edema, neutrophil infiltration and myeloperoxidase; decrease in pro-inflammatory cytokines and increase in KGF, PGE2 and IL-10;	[35]
UC-MSC-derived EVs	*E. coli*-induced rat lung injury	Increased survival	Enhanced phagocytosis of *E. coli*	[36]
BM-MSCs and BM-MSC-derived CM	Rat lung Injury	Attenuation of lung injury	Reduced levels of pro-inflammatory cytokine	[37]
BM-MSCs and BM-MSC-derived CM	Ventilator-induced rat lung injury	Reduction in injury and improvement in recovery	Reduced levels of edema, neutrophil, and alveolar IL-6 concentrations	[38]
BM-MSC-derived CM	Rat lung IRI	Protection against lung IRI	Decrease in both pro-inflammatory cytokines and infiltrating inflammatory cells, and increase in both M2-like macrophages and regulatory T cells	[39]
AdMSC-derived CM	LPS-induced mouse lung injury	Reduction in ARDS indices	Reduced endothelial barrier hyperpermeability and activation of pro-inflammatory and pro-apoptotic pathways in endothelium.	[40]
AMSC-derived CM	In vitro model of human lung IRI	Attenuation of IRI effects by improving the efficacy of in vitro EVLP	Increase in anti-inflammatory factors and up-regulation of anti-apoptotic factors	[41]
BM-MSCs and AdMSC-derived CM	Rat and human alveolar epithelial cell injury	Decreased cell injury	Decrease in pro-inflammatory factors and increase in anti-inflammatory factors; inhibition of p38 MAPK and translocation of Bcl-2 to the nucleus; Increased expression of cytoprotective glucose-regulated proteins	[43]
MSCs	Swine lung IRI	Attenuation of ischemic injury in donor lungs during EVLP and attenuation of IRI after transplantation	Increased levels of HGF and IL-4 and decreased levels of TNFα and cell death markers	[65]
BM-MSCs	HCL- and LPS-induced rat lung injury	Decreased inflammation	Decrease in proinflammatory cytokines, neutrophil infiltration, hemorrhage and interstitial edema	[122]
UC-MSCs and UC-MSC-derived EVs	Rat neonatal hyperoxic lung injuries	Attenuation of hyperoxic lung injuries	Increased alveolarization and angiogenesis; decrease in alveolar epithelial cell death, macrophages and cytokines in lung	[123]
BM-MSC-derived EVs	Mouse pulmonary arterial hypertension	Reduction in pulmonary vascular remodeling and right ventricle hypertrophy	Increased levels of anti-inflammatory and anti-proliferative miRs including miRs-34a,-122,-124, and -127.	[124]
BM-MSCs	Rat lung IRI	Attenuation of lung pathologic injury	Reduced myeloperoxidase production, decreased levels of of pro-inflammatory cytokine and cell apoptosis in lung tissue	[125]
BM-MSCs	*E. coli*-induced rat pneumonia	Reduction in lung injury; improvement in survival; reduction in lung bacterial load and suppression of inflammation	Enhanced macrophage phagocytic capacity and increase in lung and systemic concentrations of the antimicrobial peptide LL37	[126]
BM-MSCs	Hyperoxia-induced rat lung injury	Mitigation of emphysema	Increased number of alveoli and decrease in α-SMA expression by myofibroblasts	[127]
BM-MSCs and BM-MSC-derived CM	Cigarette-smoke-induced rat emphysema	Alleviation of emphysema and increase in the number of small pulmonary vessels	Decrease in pulmonary artery medial wall thickness and reduction in apoptosis in lungs with emphysema	[128]
BM-MSCs and BM-MSC-derived CM	LPS-induced mouse lung injury	Resolution of lung injury by attenuating lung inflammation	Decrease in neutrophils and increase in M2 in BAL	[129]
BM-MSCs and BM-MSC-derived CM	Mouse chronic obstructive pulmonary disease	Reduction in injury	Reduced levels of inflammation, fibrosis and apoptotic and increased production of HGF	[130]
AdMSC-derived EVs	Elastase-induced mouse emphysema	Reduction in lung emphysema	Increased levels of FGF2	[131]
BM-MSCs	Bleomycin-induced rat pulmonary fibrosis	Decreased fibrosis	Attenuation of NRF2, NQO1, HO-1, γ-GCS, lipid peroxidation, and increase in SOD activity	[132]
UC-MSCs	Rat lung IRI	Reduction in Oxidative stress damage and inflammation	Reduced levels of MPO activity and neutrophil markers; reduction in reactive oxygen species production	[133]
AdMSCs and AdMSC-derived CM	Sulfur mustard-induced mouse lung injury	Reduction in progressive histopathologic changes in the lung	Reducd levels of both M1 and M2 cells, TNF-α and IL-1β	[134]
BM-MSC-derived CM	Bleomycin-induced rat pulmonary fibrosis	Protection against lung fibrosis	Decrease in lung inflammation, fibrotic scores, collagen deposition, and cell apoptosis	[135]
SHEDs and SHED-derived CM	Bleomycin-induced mouse pulmonary fibrosis	Attenuation of lung injury and improvement in survival rate	Reduced levels of pro-inflammatory factors and increased levels of anti-inflammatory factors and M2 cells	[136]
BM-MSCs	Swine lung transplantation	Improvement in dynamic lung compliance	Reduced intrapulmonary edema	[137]
BM-MSC-derived EVs	*E. Coli*-induced mouse lung Injury	Reduction in lung edema and inflammation	Decrease in lung protein permeability, neutrophils and macrophage inflammatory protein-2 levels in the BAL fluid; increase in KGF in BAL	[138]
AdMSCs	Rat lung IRI	Attenuation of lung damage after IRI	Suppression of oxidative stress and inflammatory reaction	[139]
UC-MSCs	Swine lung IRI	Attenuation of IRI by improving the efficacy of EVLP	Increased levels of VEGF and decreased concentration of circulating IL-8	[140]
BM-MSCs	Human lung IRI and EVLP	Decreased cold ischemic injury	Decrease in pro-inflammatory cytokines and increase in anti-inflammatory cytokines	[30]
BM-MSCs and BM-MSC-derived CM	*E. coli*-induced human lung injury	Increase in alveolar fluid clearance in lungs during EVLP	KGF secretion	[31]
BM-MSCs	Human lungs rejected for transplantation and subjected to prolonged ischemic time	Restoration of alveolar fluid clearance	KGF secretion	[32]
BM-MSC-derived EVs	Human lungs rejected for transplantation	Increase in alveolar fluid clearance in donor lungs during EVLP	Improved airway and hemodynamic parameters	[141]
AdMSCs	Clinical trial	Attenuation of IRI and host immunological reaction towards the graft	Not determined	NCT04714801
BM-MSCs	Clinical trial	Attenuation of graft rejection and bronchiolitis obliteran syndrome (BOS)	Not determined	NCT02181712

MSCs: mesenchymal stromal/stem cells; BM-MSCs: bone-marrow-derived MSCs; AMSCs: amnion-derived MSCs; UC-MSCs: umbilical-cord-derived MSCs; AdMSCs: adipose-derived MSCs; SHEDs: stromal/stem cells from human exfoliated deciduous teeth; IRI: ischemia-reperfusion injury; EVLP: ex vivo lung perfusion; CM: conditioned medium; EVs: extracellular vesicles; BAL: bronchoalveolar lavage; ARDS: acute respiratory distress syndrome.
